# Book Reviews

**Published:** 1828-12

**Authors:** 


### CRITICAL ANALYSES.

Quæ laudanda forent, et quæ culpanda, vicissim

Illa, prius, cretâ; mox hæc, carbone, *notamus*.—PERSIUS.

*Commentaries on the Causes, Forms, Symptoms, and Treatment, Moral and Medical, of Insanity.* By GEORGE MAN BURROWS, M.D. Member of the Royal College of Physicians of London, &c.—8vo. pp. 716. Thos. and Geo. Underwood, London, 1828.

(Continued from p. 462.)

COMMENTARY III. *Physical Causes.*—Dr. BURROWS believes that the greatest obstacle to the knowledge of the pathology of insanity, has been the long prevailing error of studying the mental, to the neglect of those corporeal phenomena which are almost always cognizable. The hallucinations of the mind are only the signs of its disorder, as symptoms are of corporeal disorders, and are therefore of but secondary importance in the study of insanity.

"Where is the utility of studying the characters of the mental delusion? Can it signify whether a lunatic fancy himself to be a deity or a monarch, a philosopher or a conjurer, or any thing animate or inanimate? Ought we not to prefer examining the various signs which indicate functional or structural lesion, and endeavour to find out whether the attendant delirium is idiopathic, symptomatic, or sympathetic?" (P. 58.)

Dr. Burrows touches slightly, and with candour, upon the subject of phrenology. He does not presumptuously venture to assert that the subject is altogether one of error. The grounds on which it is founded demand a long, careful, and personal investigation; and he confesses that he has not in every respect given it the necessary consideration. He relates the following anecdote; but whether it proves an error in judgment of the celebrated founder of the system, or that the heads examined were examples of perverse configuration, he does not venture to decide.

"When Dr. Gall was in this country, he went in company with Dr. H. to visit the *studio* of the eminent sculptor, Chantrey.

"Mr. C. being at the moment engaged, they amused themselves in viewing the various efforts of his skill. Dr. Gall was requested to say, from the organs exhibited in a certain bust, what was the predominant propensity or faculty of the individual. He pronounced the original must be a great poet. His attention was directed to a second bust. He declared the latter to be that of a great mathematician. The first was the bust of Troughton, the eminent mathematician; and the second that of Sir Walter Scott!

Lavater made a similar blunder in physiognomy. He pronounced Kant to be a thief, and a thief to be a philosopher

"Talent, the phrenologist asserts, is relative with the ample development of the cerebral mass. Mr. Chantrey exhibited to Dr. Gall drawings of numerous heads. The cranioscopist selected one, whose ample cerebral development gave a sure index of vast talent. It was a fac-simile of the head of the Earl of P—mf—t!" (P. 67, *note*.)

Dr. Burrows has himself assisted at several accurate anatomical investigations, conducted by eminent demonstrators, of the crania of insane patients who had been under his care, and who had exhibited, up to the hour of their decease, the most furious symptoms of mania for months, and yet not a vestige of disease could be traced.

"Similar results have strengthened the assumption that insanity is purely a mental affection: hence also the impression that, whenever there was any lesion or disease observed in the encephalon, such was the effect, and not the cause, of the insanity. And this inference was further confirmed by the remarks of Pinel, Esquirol, &c. that the same morbid appearances displayed in the bodies of those who had died mad, were discovered in the corpses of those who had died of diseases quite unconnected with insanity. Others were weak enough to imagine, because many insane persons recovered their faculties, that therefore their derangement could not have a corporeal origin.

"But we ought not to presume, because there are no visible marks of a morbid condition of the brain or its appendages, that therefore the whole are in a perfectly healthy state. Where is the anatomist who will dare maintain that a brain is free from disease or structural change, because, after the most minute inspection, he cannot discover any?

"That eminent physiologist, Haller, conceived that, 'in disorders of the mind, the brain and its connexions are usually affected: and when, in some rare instances, we can discover no disease of these parts, we may conclude, either that it is seated in their very elementary particles, or has not been sought for with sufficient patience and attention.' But, even in the elementary composition of the brain, anatomists are not agreed. How, therefore, can they pronounce whether a brain is in a sound state or not?

"Except in connate idiocy, and mental derangement from mechanical injuries, what direct proof is there that insanity has its seat in the brain?" (P. 70.)

When an insane person has been cut off by an acute disease or accident, or has destroyed himself, and the insanity has been of short duration, there are seldom exhibited any alterations or morbid appearances in the encephalon, beyond slight vascular congestions or effusions. In long standing cases, a post-mortem examination *generally* exhibits strong evidence of disease in that organ. Sometimes, however, violent insanity has continued for years, and not a visible trace of diseased structure or action has been discovered in the brain or elsewhere. The same fact holds good with respect to many other diseases.

"Is not the human body subject to, and influenced by, peculiar diatheses? Will the scalpel detect the apoplectic, hydrocephalic, scrofulous, or gouty diathesis? The testimony of Morgagni, Cheyne, Abercrombie, Powell, Stark, &c. prove that death from apoplexy frequently occurs; and yet no evidence of cerebral lesion has been discovered on the minutest investigation of the brain." (P. 72.)

The author very properly asks, why should we expect to discover by the eye the maniacal diathesis, when all others are impenetrable?

"Again: the organs of the external senses are five distinct pairs of nerves. But although the offices of these nerves be distinct, having no relation one to the other, yet there is no difference in their internal structure, by which their appropriate functions are manifested. It is not by examining the substance of any detached nerve, that we ascertain the specific sense which it conveys: that is known by its situation and connexions, and not by its texture.

"Further: who can say that excitement, nay inflammation itself, has not existed in the brain, if the circulation through a great portion of it is, as it is said to be, colourless? A morbid action of other important organs is indicated by appropriate symptoms, and yet, on a subsequent examination of the suspected organ, it often appears perfectly sound. A disease of an organ may be structural or functional; and the former may cease, while the latter, though a consequence, is long protracted. Thus, as every other organ imbibes morbid actions, of which post-mortem examinations offer no evidence, and which morbid action is *sui generis*, is it inconsistent to conclude that the brain also assumes a peculiar action, which may be appropriately designated the maniacal?" (P. 73.)

The collection made by Dr. Scipion Pinel, embracing those of Messrs. Esquirol, Villermai, Beauvais, and Schwilgaé, and which in the aggregate comprise 259 dissections of maniacs, offers a mass of pathological examinations particularly worthy of record. They are thus classed:

"*Lesions of the Brain.*

| | | |
|---|---|---|
| Apoplexy . . . . . | 27 | |
| Organic lesion of the substance of the brain | 19 | |
| Organic chronic lesion of the membranes . | 22 | |
| | | — 68 |

"*Lesions of other Organs.*

| | |
|---|---|
| Chronic peripneumony . . | 20 |
| Phthisis . . . . | 22 |
| Chronic peritonitis . . | 9 |
| Chronic pleuritis . . . | 7 |
| Chronic inflammation of the digestive canal | 50 |
| Other organic lesions of this viscus . | 13 |
| Lesions of the liver . . | 5 |
| ——— kidneys . . . | 3 |
| ——— ovaries . . | 2 |
| ——— uterus . . . | 4 |
| | —135 |
| | 203." |

In the other fifty-six corpses, there was no visible evidence of disease in any of the viscera of the three great cavities. The spinal cord was examined in only two cases.

"From these dissections it follows: 1. That lesions of the brain, the organ of the intellectual functions, are in the proportion of one to two of those of the other viscera; 2. That more than one in five corpses of maniacs present no evidence of any disease whatever! 3. That, in a great majority of cases, the insanity was a sympathetic affection; and 4. That as, in more than a fifth of 259 dissections, no lesion or alteration could be detected, it strongly corroborates the opinion that, when such lesions or alterations are observed, they are posterior, and not anterior, to the development of the mental derangement." (P. 75.)

But, from whatever predisponent cause insanity may proceed, if it be not primarily an organic affection of the brain, it ends in being so. The author, though somewhat sceptical upon the subject, is still willing to concede that the evidence of Bayle upon the pathological appearances detected in the brains of insane patients is respectable. Even this cold mark of confidence seems to proceed rather from politeness than sincerity; for in the next page Dr. Burrows remarks, "From his rapid career," (still speaking of Bayle,) "I fear a vivid imagination and fondness for theory are leading his judgment astray, and that, like the patients whose bodies he examines, he adopts illusions for realities." For our own parts, we candidly confess that *we* can place but little confidence in distinctions, which are established upon the supposition that it is possible to assign one set of symptoms to inflammation of the arachnoid membrane alone, and another in case the pia mater is affected conjointly with the arachnoid.

Although Dr. Burrows differs from the opinions of Vogel, Dumas, &c. that mania always proceeds from an inflammatory state of the encephalon, yet he does not doubt that mental derangement is often the sequel of inflammation of the brain. That insanity is the effect of cerebral inflammation, he is persuaded is an error as dangerous as it is common. In his opinion, nothing is more clear than that the inflammatory and maniacal actions are totally distinct. He who will reflect upon the general condition of the patient,—upon the symptoms frequently exhibited during mania,—and upon the inefficiency, and even mischievous effects which arise but too commonly from the heedless and indiscriminate application of a mode of treatment destined merely to check inflammatory action, must, we apprehend, agree with Dr. Burrows in the doctrine he maintains upon this point.

The various hypotheses that have been suggested to prove that insanity is a disorder of the "peculiar and subtile fluid of the nerves," are briefly considered. Dr. Burrows very wisely does not enter into the question of what may be the nature of the nervous power, or how it acts and connects us with the external world. But he considers it essential to deny that the proximate cause of delirium and insanity arises from a diseased action of the fine vessels which secrete and circulate the nervous fluid.

"It is to be lamented, when the doctrine of the secretion and operation of this fine and subtile nervous fluid circulating through the brain is exploded, that it should be revived as the true physiology of the organ of intelligence, without any new evidence or argument in support of it. Dr. F. Willis adopts the obsolete opinions of Cullen, Crichton, and Good, to prove the correctness of his own postulate, viz. that insanity depends on 'a specific diseased action of those fine vessels that secrete the nervous fluid of the brain;' but he is silent on the testimony of subsequent physiologists, who disprove, by experiment, the existence of this nervous fluid." (P. 87.)

Although we know not the causes nor the mode by which sympathies act, yet we have abundant proof of their operation in originating diseases which reciprocally act on the mind.

"There is no organ with the morbid actions of which the functions of the brain so frequently sympathise as the liver. As the connexion is intimate, so is it reciprocal; for morbid actions of the former equally, and perhaps as frequently, disturb the functions of the latter. In importance, the functions of this organ are only second to those of the brain, as far as regards the operations of health; and as in the brain, so too in the liver, the circulation of the blood is complex, and very liable to be interrupted by extrinsic causes. Hence the greater facility of disturbing its functions.

"All the passions, anger especially, violently affecting the sensorium, act immediately on the liver; and every excess that disturbs the functions of the stomach easily determines blood in undue proportion to the vena portarum, where, on account of the remoteness of this vessel from the heart, the motion of the blood is always sluggish, and therefore congestion is easily induced. The bile, consequently, is secreted in scanty quantities, the alimentary processes become ineffective, a morbid action of the connecting nerves follows, and the functions of the brain are implicated and disordered.

"Many facts attest that blows on the head will create, not simply disordered function, but disorganization of the liver; and, vice versâ, nothing is more common than instances of mental disturbance originating in injuries of this organ, or in secretions of morbid bile, or obstructions of the biliary ducts by gall-stones, spasm, &c.

"Diseases of the hepatic system will even originate delirium, furious mania, melancholy, and suicide.

"Insanity is much more common among the lowest classes than the supporters of its mental origin are inclined to admit. Now, drunkenness is certainly the great vice of this class in Great Britain and Ireland, and the propensity is gratified usually by ardent spirits. In a table of 1370 lunatics admitted into the Asylum at Cork, Dr. Hallaran says 160 were insane from this unhappy indulgence." (P. 93.)

A morbid condition of the chylopoietic viscera is, sympathetically, a frequent cause of mental derangement.

"Gastric irritation, too, is a much more frequent cause of mental derangement, through this mysterious agency, than is usually imagined. Long-continued nausea is often a precursor of a paroxysm of insanity. Violent nausea, also, from sea-sickness, continued for a few hours, has produced mania in three instances within my knowledge." (P. 95.)

Dr. Burrows has known insanity to arise, in two instances, from the irritation of cutting the dentes sapientiæ.

Commentary IV. contains many interesting observations upon the subject of hereditary predisposition, which too frequent experience instructs us is a prominent cause of mental derangement. Esquirol assigns only 150 out of 264 cases in his private practice to *hérédité*. Dr. Burrows has clearly ascertained that an hereditary predisposition existed in six-sevenths of the whole of his patients.

Commentary V. *on the Vascular and Nervous Systems.*—In this section it is the object of the author to examine if there be not ground to assume that the disorders of the sanguiferous system have as great, or greater, influence in originating insanity, than those of the nervous system. Dr. Parry, in his Elements of Pathology, has very ably commented upon this subject. But, unfortunately, this highly talented author almost totally neglects the influence of the nervous system upon the general economy. He states, indeed, in his preface, that "he utterly and for ever disclaims all reliance on the neurological systems of pathology hitherto extant." The practitioner, however, must neither entirely neglect the doctrines which are founded upon these systems, nor must he yield implicit confidence to those which maintain that the first encroachments of disease are to be sought for in the vascular apparatus alone. A very moderate knowledge of the general laws of the animal economy must convince us of the fact, that—

"Neither the vascular nor the nervous system can receive an insulated impression; for whether the irritation which an impression produces be applied either to the sanguiferous or lymphatic vessels, or the branches of nerves in connexion with them, both systems participate, and equally suffer. Thus we find alterations, or even disorganizations of structure, result from long-continued nervous complaints: and at length the parts so affected proceed to a state of erythism or sub-inflammation; and, finally, to the highest degree of excitement, or real inflammatinn." (P. 111.)

In most of the ancient authors we find observations, implying that irregularities occur in the sanguiferous system in all cerebral affections: and experiment and observation have proved that, although the sanguiferous and nervous systems are, as to their inherent powers, independent of each other, yet that they are intimately associated, and exert a mutual influence.

"All the precursory symptoms, as well as those on the actual access of mania, characterise a disorder in the circulation: as headache, or sense of distention; throbbing of the arteries supplying the head; tinnitus aurium; the eyes bloodshot, often prominent and shining; flushed face, and preternatural degree of heat of the scalp; while the extremities are cold, pulse hurried, &c. In some, these signs of accelerated circulation long continue; in others, they are observable only on the approach of a paroxysm, and gradually decline with it.

"A state of the congestion of the capillary and venous system is a consequence generally of too great determination; and is commonly conspicuous on dissection of the insane." (P. 116.)

It is important to remember that the irritability of a part is in a ratio with its vascularity. The brain must therefore be highly susceptible.

Commentary VI. *Disorders of the Circulation.*—There are two morbid conditions of the circulation, which, though directly opposed, immediately influence the intellectual functions, and which, whenever they exist, are manifest and frequent causes of deranged intellect. First, when the blood is in quantity or momentum excessive; and, second, when the blood is in quantity or momentum defective. We would observe, for the benefit of those who are as yet upon the threshold of their professional avocations, that the latter condition of the vascular system claims as much of their attention as the former. It is too much the fashion of the modern school of pathology to instil into the minds of students that most, or perhaps all, diseases are to be attributed to increased momentum of blood.

Dr. Burrows confirms the fact mentioned by Mason Cox, that there generally exists a degree of plethora about the heads of maniacs, and frequently when other parts of the system are in a state of exhaustion and debility. The experiments of Dr. Parry, by compressing the carotid arteries in various cerebral disorders, and of suspending the paroxysms of mania as long as the pressure was continued, prove the influence that the afflux of blood exerts upon the intellectual faculties. The good effects of tying the carotid artery on the side in pain, in the ordinary headache attended with throbbing of the carotids, has since been shewn by Sir Astley Cooper and Mr. Travers; and the results in some degree confirm those of Parry. In some recent cases of mania, attended by severe headache and evident determination of blood to the head, while pressure was continued on the carotids, Dr. Burrows found there was a partial suspension of the symptoms. In one young woman, liable to sudden and furious maniacal paroxysms, if the coming-on of the premonitory pulsation of the carotids were watched, and pressure by the thumb was applied, the paroxysm was either prevented or moderated.

In this and the succeeding Commentary, Dr. Burrows points out the various morbid conditions of the sanguiferous system, and their influence on the cerebral functions.

*Hemorrhagic discharges* form the subject of the eighth Commentary. From the age of Hippocrates to the present, physicians have attached much importance in the cure of all cephalic affections, and of melancholia particularly, to hemorrhagic discharges from piles. The author can easily conceive that, in a constitution subject to periodical discharges of blood from the hemorrhoidal vessels, to suppress such discharge might sensibly affect the brain and disturb its functions, but he has never had any direct proof of insanity being induced from this cause.

"The opinion that a discharge of blood from piles often proves critical and removes insanity, I have never seen confirmed. From the frequent reference to this disease by foreign writers, we may imagine that it is much more common abroad than in this country; and, if so, a wider field of experience may have offered examples of the cure of mental affections by a natural or artificial discharge of blood from the hemorrhoidal vessels, which does not present itself to British practitioners." (P. 149.)

It is of much importance to remember that "hemorrhagic fluxes, however, are not always proofs of plethora, or even of turgescence of the sanguiferous system. Sometimes they import a deficiency of blood, or an attenuated condition of that fluid, from the effects of which a state of atony and fatuity may follow."

In the ninth Commentary, the author briefly refers to various diseases which are commonly complicated with insanity, as vertigo, epilepsy, convulsions, apoplexy, paralysis, &c. From the general view he has taken of the physical phenomena of disordered intellect, apparent in the living and the dead, Dr. Burrows infers—

"1. That the circulating system, in every case of insanity, is morbidly, though often differently, affected.

"2. That the healthy exercise of the intellectual functions is dependent on a due regularity in the supply and momentum of blood to the brain, the source of the nervous system.

"3. That, while the vascular and nervous systems act in concert, the harmony of the intellectual functions is undisturbed.

"4. That, in all cases of insanity, the vascular and nervous systems are in a state of opposition.

"5. That, in incipient insanity, excitement of the vascular system generally predominates; in chronic insanity, the nervous.

"6. That, in all the diseases complicated with insanity, there is a well-marked ascendancy of either system.

"7. That, as the actions of the two systems approximate, improvement in the intellectual functions takes place; and that, when they again act in unison, sanity is reestablished." (P. 201.)

Commentary X.—The author has hitherto treated only of those disorders which have a fixed and tangible character: in which there is a visible disturbance both of the mental and corporeal functions. Mental derangement, however, is frequently produced in a more indirect and mysterious manner. Derangements of remote parts frequently occasion mental disturbance, by shifting morbid actions to the brain.

"There are three modes through which the brain is morbidly affected by remote diseases, and insanity is induced.

"1. By metastasis, or translation;

"2. By sympathy;

"3. By conversion.

"These three modes of originating mental derangements have hitherto been usually considered as one and the same, under the designation of sympathetic causes, and hence some confusion has arisen; but, in fact, they are distinct morbid actions.

"In metastasis, the part or texture primarily affected is completely freed when the morbid action originating in that part removes itself to another; but the morbid action may return again to its original seat, and leave the part secondarily affected free.

"In sympathy, the functions of an organ or part primarily deranged may remain permanently so, while those of a remote organ or part shall assume all the characters of the morbid action of the primary affection.

"In conversion, a disease shall leave a part or texture where it was situated, and another disease in some other part or texture is immediately superinduced." (P. 203.)

All morbid actions are susceptible of different modifications, and, when transferred to the brain, occasion different degrees and forms of delirium.

"The morbid action of organic diseases is often suddenly transferred to the brain, and occasions, while it lasts, a real delirium, to the complete suspension of the original disease; and the patient, from the last extremity of existence, becomes suddenly endowed with a degree of muscular power truly amazing. In this condition all the phenomena of insanity are developed. It may be continued for months; and, upon the sudden subsidence of the delirium, the original disease resumes its course, and the patient dies in a few hours or days. This frequently occurs in pulmonary consumption.

"Aretæus notices the propensity in phthisis to induce insanity; and Mead has observed, that there appears an interchangeable relation between lunacy and phthisis pulmonalis; the latter being cured by the accession of the former, and recurring as soon as the brain resumes its natural functions.

"In the last stage of consumption, a delirium is apt to come on; and I have several times been called to visit a patient in this state, from an impression in his own opinion that he was insane. But I have invariably found this symptom a certain indication of approaching death." (P. 205.)

The result of our observations would lead us to doubt the *frequent* occurrence of insanity during the progress of pulmonary consumption. On the contrary, we believe that consumptive patients usually retain full possession of their mental powers until the last moments of their existence. The temporary delirium that occasionally arises during the continuance of a high state of hectic fever is totally distinct from insanity; and this is the only affection of the mind which we have witnessed in pulmonary consumption.

One interesting case has occurred to us, in which insanity proceeded from the rash suppression of chronic diarrhœa. It should be observed, however, that opium was the remedy employed. Dr. Burrows relates the following analogous example:

"A gentleman, aged seventy, of a very delicate constitution and most temperate habits, had for two or three years, notwithstanding the best medical advice and the most careful conduct, been subject to constant colliquative diarrhœa.

"At length he was so reduced as to be given over, and his death was hourly expected. By way of affording him present comfort, a much larger dose of opium than he had ever taken, mixed with a powerful astringent, was given him. It effectually stopped the purging. He took plenty of nutriment, and gradually recovered his strength; but, as he grew stronger, a total change in his moral character was observed. I had known him many years. His disposition, before even, meek, and remarkably correct and modest, became turbulent, noisy, extravagant, and obscene; and he laboured under the most extraordinary and ludicrous delusions. He lived seven years in this changed condition. At length his bowels became very lax again, and he gradually wasted from the effects of it. But probably the morbid action of the brain had been so long continued as to produce some organic change, for the character of his delirium was unaltered till his last breath. He died in my presence, humming the tune of an old ballad." (P. 209.)

In this case, the author observes, "that the morbid irritation of the bowels was evidently transferred to the brain." It is not, we think, improbable that the large dose of opium which was exhibited had also some share in causing the mental derangement.

It is a well-established physiological fact, that a primary disease or injury of the brain may induce affections of other organs; and, on the contrary, that the brain may be morbidly affected by a diseased action of remote parts.

"In adverting to the causes of insanity, the possibility of such remote and obscure actions should always be borne in mind; for these reciprocal sympathies have a most powerful and extensive influence in originating and transferring morbid actions; and whether the intellectual derangement be primary or secondary, it is of the utmost importance to ascertain.

"The origin and affinities of morbid sympathies are the most obscure of nature's operations; and, like the essence of the intelligent principle, still, and for ever probably will, remain enveloped in mystery. Inscrutable, however, as the sources and connexions of these affections may be, still, as insanity is frequently to be traced to the latent influence they exercise on the human system, the doctrine and effects of sympathy claim ampler notice." (P. 210.)

Abscesses of the liver are noticed by many authors as consequent on injuries of the head. The passage of gall-stones is sometimes attended with a long delirium, which, from its continuance, might be mistaken for insanity.

"About nine years ago, the wife of a medical friend continued for two months in a state of delirium so entire that a ray of reason did not break in. There was great restlessness, with quick pulse, dry skin; the tongue dry, brown, and furred; and she became much emaciated. When reason began to dawn, she expressed an earnest desire to be left alone that night, as she was convinced, if not disturbed by others, she should have some sound rest. Her request was complied with, and she slept uninterruptedly fifteen hours. She awoke amazingly refreshed; and from that moment her mental and bodily health rapidly recovered. She then went into the country, where she remained two months, and returned home lusty, and in excellent health. Three days after she returned she was seized with vomiting, which lasted for many hours. On the following day she took an aperient, during the operation of which she experienced a distinct sensation, as if something had given way in her right side. The succeeding morning she had a natural evacuation of the bowels; and, upon examining the contents, she discovered two singularly large gall-stones. These I examined minutely: one weighed three drachms, the circumference was three inches, and the length one inch and a quarter; the other weighed ninety-two grains and a half, and the circumference was two inches and a half, and the length one inch and an eighth. She has since experienced no return of her complaints.

"The mental derangement in this case was probably kept up during the two months, when there were symptoms indicative of the inflammation which the passing of such comparatively large masses must have occasioned.

"As delirium supervened on the commencement of the attack, she was of course incapable of accurately describing the seat of her sufferings. The cause, therefore, was not suspected, till discovered by the patient's own sensations, and examination of her dejections.

"This case forcibly illustrates the necessity of watching most minutely every sign and expression indicative of bodily pain, as well as of disordered function, in cases attended by delirium." (P. 213.)

Furious madness is sometimes produced by irritation mechanically applied to a remote part. Hufeland mentions a boy, between thirteen and fourteen years of age, who suddenly began to talk in a very wild and incoherent way, and at length became ungovernable,

"This state was assuaged by soporifics. But the paroxysm was observed to recur whenever he was placed on his feet. On examination, a reddish spot was noticed in one foot, which, when pressed, always occasioned a fresh paroxysm. Upon an incision being made, a minute piece of glass was discovered and extracted. During the operation the patient was furious; but every symptom of violence vanished when the offending cause was removed." (P. 215.)

*Conversion.*—The diseases which frequently interchange with insanity, are those in which the circulation is obviously disordered; as in various forms of idiopathic fever, apoplexy, hemiplegia, epilepsy, convulsions, cephalic and comatose affections, hemorrhagic and hydropic complaints, gout, rheumatism, phthisis, asthma, &c. All these diseases may alternate with mania, or with each other.

The opinion has generally prevailed that lunatics are insensible to atmospheric changes and impressions. Dr. Burrows correctly maintains that experience proves that such patients are by no means insusceptible of the extremes of temperature. He observes—

"I confess it is quite wonderful to me how it could have escaped observation, as I suppose it must, that lunatics, especially melancholics, are commonly subject to an extremely languid circulation in the lower extremities; and therefore, without extraordinary care, must of course suffer greatly from cold.

"Extremes of heat and cold are not only in themselves causes of insanity, but sensibly affect both the bodily and mental state of most lunatics. In whatever asylum the patients are treated under the conviction that they are insensible of cold, there, assuredly, mortification of the extremities will be common. In most of the British lunatic asylums which I have inspected since the year 1821, I found proper care taken to prevent injury from cold. Indeed, I had frequent occasion to remark that the day-rooms were sometimes too much heated; so that, when the patients went from those rooms into the galleries and airing grounds, the atmospheric change was too great; and hence catarrhal, ophthalmic, and rheumatic affections were induced. But in others the patients were by no means sufficiently protected from the effects of cold, and, as might be expected, I there found mortification of the extremities less rare." (P. 230.)

Mortification of the extremities, however, is not always the simple effect of exposure to cold, or of neglect. The author has met with instances of it in private practice, which he has attributed to that diminution of the vital principle which he has noticed to be so often conspicuous in the constitution of persons in whom the influence of the intelligent principle is deteriorated. He quotes two examples.

In the thirteenth Commentary, Dr. Burrows offers a few observations upon the influence of climate, occupation, sex, and age, on insanity. He mentions many facts which induce him to conclude that it is not climate, but a high temperature, which disposes the intellectual functions to derangement.

We now arrive at the second part of the volume; the first section of which treats *of the Division of Insanity*. The definition of the various forms of insanity is a tempting inducement for an author to enter into fanciful, and, as far as regards practical purposes, useless speculations. Dr. Burrows, however, is not to be led away from the purpose he professes. Practical improvement is the object of his work. Definitions of the morbid phenomena of mind he would leave to schoolmen, who love to indulge in subtleties. He confines himself to a simple division of the most conspicuous and ordinary forms in which insanity appears, and he considers that the best rule for every body to observe, when attempting to form a judgment on any particular case of insanity, is to take care and preserve his own faculties clear, and as free from the mysticism of speculative philosophy as from the trammels of nosology. He might have added, that it is the duty of the practitioner to approach the consideration of every other disease with the same free and unshackled spirit of inquiry. The efficacy of our practice can never depend upon our knowledge of the abstract, and too often fanciful, arrangements of diseases which have been adopted by nosologists. For the convenience of discussion, however, as well as for practical purposes, some arrangement must be followed. Dr. Burrows considers Esquirol's the least objectionable, because the most unpretending and simple.

"But it is defective; since it omits delirium and hypochondriasis, which, in my judgment, have better claims to be considered as distinct species than mania and melancholia. It is true, if delirium be received only in its ordinary acceptation as symbolical of intellectual disorder, it does not merit the rank of a distinct malady. But I think that there is ground to consider it as a frequent idiopathic affection, though certainly much more generally as sympathetic, and often as symptomatic. This point, however, I shall discuss more at length when treating on delirium.

"The order I shall adopt, therefore, is, I. Insanity. 1. Delirium; delirium tremens. 2. Mania; puerperal insanity. 3. Melancholia; suicide. 4. Hypochondriasis. 5. Demency. 6. Idiocy. (P. 258.)

Commentary II. *Character of Insanity.*—In the common acceptation, that person is insane or mad, or in a delirium, when any single, or several faculties which synthetically constitute the mind, exhibit signs of disordered function. Madness, says SAUVAGES, is the dream of him who is awake; and—

"This waking dream may consist in an unnatural rapidity of thoughts, or in a morbid association of them with some known or recollected object, or in the substitution of illusions for realities. Sometimes perception is correct, but memory and judgment are defective; or the reverse may obtain.

"Sometimes sensation and volition are equally affected: one, or several, of the external senses shall be perfect, and the others changed; and sometimes all are implicated, or the mind and the will may be at variance. Every sensation, thought, or idea, may have place; but have neither order, object, connexion, or stability. Neither, though they perceive and think, can the insane always connect, compare, or abstract. These must be received as general propositions, but with exceptions. For instance, the faculty of associating their ideas with words and things, and of applying them to their own situations, so as to combine and execute plans, is sometimes exhibited in a manner most correct and wonderful. Often the most trivial thing will induce certain ideas, which at their birth are correct; but, the judgment being leesed or perverted, the catenation is broken, their application mistaken, and the most wild and incoherent thoughts, expressions, and actions follow.

"Hence impressions may be either strong or weak: the mind in the one case pertinaciously adhering to one illusion, to the exclusion of every other idea; in the other, the delusive impression is so evanescent as to induce us to presume that the memory is impaired; and yet hereafter the last idea may revive, and recur with vivid force.

"Sometimes the attention to internal feelings supersedes that to all external objects, or the reverse; or one faculty may acquire such an excessive acuteness, while the others retain their natural condition, that such a preponderance of the trains of thought and actions connected with the objects of that sense ensues, as constitutes insanity." (P. 261.)

One or several of the exterior senses are usually obtunded, perverted, depraved, or alienated. The sense of hearing usually suffers the first and most. Melancholics are sometimes so wholly abstracted that it is quite impossible to discover from them the nature of their hallucination, or any thing in which they ever took an interest.

PINEL refers to a variety of insanity which is very common, and which he denominates *folie raisonnante,* reasoning madness. It is marked by a propriety of ideas, and a sort of judgment very imposing.

"The patient can read, write, and reflect, as if he possessed a sound mind; and yet is at the same time capable of the most outrageous violence, or will tear every thing to pieces that comes in his way; or his insanity may shew itself simply in general profusion, in dress, and other extravagances. It is this variety of insanity, and power of self-possession, which so often deceives the inexperienced, who do not recollect that sanity or insanity of mind cannot always be detected in conversation, which is often very plausible, and apparently correct. The delirium in this case shews itself in conduct, and not in speech, and is very difficult to discover.

"Again, the same author designates another variety, *manie sans délire*, (mania without delirium,) in which there is no sensible alteration in the functions of the understanding, as perception, judgment, imagination, memory, &c.; but yet there is a blind impulse to acts of violence, or even to sanguinary fury. This state he ascribes to a perversion of the affective functions." (P. 267.)

In the opinion of Dr. Burrows, this is both an absurd and a dangerous distinction. It is absurd, because he who can perpetrate such acts, and yet be in possession of all these faculties, is neither in a delirium nor mad. It is dangerous, because any enormity might be committed, and the perpetrator plead this form of insanity, and hold himself irresponsible for his actions.

We cannot dwell upon the innumerable visions which in different cases haunt the imagination of insane patients. The symptoms of corporeal derangement which precede or accompany the state of insanity demand our utmost attention. Fever has been said by some modern authors, as Drs. Hallaran, F. Willis, &c. to be the accompaniment of mental derangement. Dr. Burrows, however, has never seen a case of pure mania or melancholia accompanied by real pyrexia, except when some other acute affection attended, of which the fever was a symptom. Lunatics are usually costive; but this is often more the effect of indolence or disregard of natural calls, than from torpor of the intestinal canal. An opposite impression has induced the exhibition of violent cathartics in almost all cases, and often to the great distress and injury of the patient. The insane are generally unconscious of their condition; sometimes, however, the contrary is the fact, but yet they are most artful in concealing it. Others confess and lament it feelingly, and still cannot repress the propensity to make irrational proposals, or do any ridiculous thing, or commit suicide or some other kind of violence.

"A lady, of good family and of most amiable character, aged forty-eight, with an hereditary predisposition, and who had been insane about twenty-five years before, became a good deal affected from a younger sister having suddenly manifested insanity. Shortly it was necessary to confine the elder to her room for the same cause. In a few days the younger sister, for want of due precaution, destroyed herself. The fact was concealed from the elder sister; but she likewise betrayed a suicidial propensity. She confessed the feeling, and reasoned upon it as an aberration of her mind, and as sinful, and entreated not to be trusted. In fact, she made many attempts on her life. She had many other delusions; one of which was, that another woman was within her, and prompted her insane ideas and actions. At times she was highly excited, and would dance on the tables and chairs, tear her linen, and bite those about her, if permitted; and, from her language, a slight degree of nymphomania might be suspected. It was quite distressing to hear the occasional expression of her deep sorrow that she was unable to control her impious and immoral feelings, and extravagant actions.

"Such cases demand the greatest care, and the kindest consideration." (P. 274.)

Insanity may be continued, remit, or intermit, but it is observed under no form or regular crisis. An effort, however, seems sometimes to be made by nature to throw off the influential cause. "We often find that the formation and discharge of an abscess, especially of scrofulous glands, the appearance of a cutaneous eruption, a fit of gout, or an hydropic effusion, will terminate long-continued mental derangement." Many other phenomena present themselves, and mark the physical and moral characters of disordered intellect. Some require particular attention, such as physiognomy, position, sensation, muscular powers, fasting, odour. Upon each of these subjects Dr. Burrows briefly comments.

[To be continued.] vol. lxi. p. 61.

---

*Observations on the Nature and Treatment of Fractures of the upper third of the Thigh-bone, and of Fractures of long standing; shewing that Fractures of the Neck of the Femur, and others which occur in the upper third of this Bone, admit of being united, so as to restore the Natural Powers of the Limb, without Deformity or Lameness; also, that the principal Cause which prevents Fractures of the long Bones from uniting is attributable to the inadequacy of the usual Modes of Treatment; and that Fractures which have existed many Months might generally be united by the proper Employment of Mechanical Means alone, with almost as much facility as simple Fractures in the recent state. Illustrated by Cases obtained from Public and Private Practice.* By JOSEPH AMESBURY, Consulting Surgeon to the Royal Union Association; Surgeon to the South London Dispensary; Lecturer on Surgery, &c.—8vo. pp. 315. Plates. T. and G. Underwood, London, 1828.

THE mechanical ingenuity which enables Mr. AMESBURY to contrive his apparatus for the treatment of different fractures, and the professional dexterity with which he applies them, have for some time been known and duly appreciated by his surgical brethren. In the present volume the author has entered, at some length, into the consideration of the nature and treatment of fractures of the upper third of the thigh-bone, and of fractures of long standing which occur in the upper and lower extremities; and it is his object to shew that deformity and non-union are more to be attributed to the imperfect modes of treatment still generally advised, than to any other cause. Many of the cases adduced to illustrate the views and treatment of the author have been conducted in public hospitals, through the kindness of the surgeons under whom they were admitted.

Mr. Amesbury has found it necessary, in the course of his inquiries, to condemn some doctrines and plans which are now much recommended and pursued. He hopes, however, he might regard the surgeon as his friend, and at the same time oppose such of his principles and practice as seem to be objectionable. We *hope* so too; for in the free statement of his opinions he does but perform an imperative duty to the public. But we much fear that not unfrequently the slightest opposition even upon practical subjects, particularly if the discrepancy of opinion be publicly stated, leads to something approaching to hostile feelings, which from such a cause ought certainly never to enter the breast of any man who is sincerely and liberally devoted to his profession, and who regards it not merely as a trade by which a certain quantity of money may be obtained, but as a philanthropic pursuit in which the general interests of humanity are concerned. We have been led into this observation from having more than once observed the hasty and discreditable resentment which has been kindled even by the most temperately stated objections to opinions upon professional subjects.

The author commences his work with a description of the causes, symptoms, and nature of fractures of the upper third of the thigh-bone. Sir Astley Cooper has divided fractures of the neck of the thigh-bone into two kinds: those within the capsular ligament, and those external to it. Mr. Amesbury proposes a subdivision of each of these kinds. Those fractures which occur entirely within the synovial capsule might be divided into fractures without any considerable laceration of the close coverings of the neck of the bone, and into fractures accompanied with an extensive laceration, or complete division of these coverings. Fractures external to the capsule might also be divided into two kinds; one of which is accompanied with little or no laceration of the investing soft parts, and the other with great laceration or complete division of them." The neck of the thigh-bone may also be partially or completely broken through. Incomplete or complete fractures may be transverse, oblique, or comminuted. The author observes, that some surgeons might be inclined to doubt the possibility of fractures occurring within the synovial capsule, without any considerable laceration of the close coverings, in consequence of their tenuity. "*From analogy*, we are led to believe that fractures of the neck of the thigh-bone within the capsule may take place without any considerable division of the periosteum and reflected membrane, because we find that fractures occur in other situations with but little laceration of the periosteum." The inference drawn from experiments after death appears to us scarcely admissible. But Mr. Amesbury appeals to the satisfactory evidence of facts. In one instance he had an opportunity of seeing a fracture of this kind, the patient having died from organic disease a short time after the accident.

In old people, fractures of the neck of the thigh-bone occur from very slight causes, and the diagnosis of such injury, when unaccompanied with laceration, is extremely difficult. As far as Mr. A. has been able to determine, the symptoms which accompany these injuries are few, and by no means prominent.

"Notwithstanding the existence of a fracture of this description, the patient might be able to exert considerable power in the limb. He might be able to bend it upon the pelvis, or to roll it inward, immediately after the accident: not, however, without giving himself pain. There is but little or no shortening of the limb. The foot may or may not be everted. We may or may not be able to elicit crepitus. Hence it will appear that the two most striking symptoms which accompany a fracture of the cervix femoris with laceration do not exist, or at most very slightly, while the close coverings remain entire, and the neck of the bone continues of its natural length. The reason of this will immediately appear, when we consider that the retentive power of the periosteum and reflected membrane being entire, or nearly so, prevents the fractured ends from being much displaced, and at the same time, in a very great measure, prevents eversion of the foot; and, in consequence of the great facility with which the head of the bone moves in the acetabulum, crepitus will seldom be produced. In order that the crepitus of fracture should be rendered evident, it is necessary that the broken surfaces should be made to rub upon each other. This cannot sometimes be done, in these accidents, without much difficulty; for the head of the bone moves readily in the acetabulum, upon receiving the slightest impulse; and this motion of the head of the bone cannot always be restrained, especially when the investing membranes are but little torn: consequently, when the shaft of the bone is moved, the head of the bone commonly moves with it simultaneously; and when this is the case, in the same relative degree, crepitation is never felt." (P. 8.)

As the symptoms of this accident are confessedly obscure, much attention must be paid to the history of the case.

"A great and sudden diminution of power in the limb, referred principally to its upper and inner part, and occurring immediately after the infliction of an injury of that description which usually produces fracture of this part, must be regarded as a symptom of considerable importance. There is tenderness in the joint, and some pain experienced in the soft parts in the direction of the pectineus muscle and the tendon of the psoas magnus and iliacus internus, and sometimes in the hollow behind the trochanter. The patient may be able to turn the limb inward or outward; he may be able to bend it upon the pelvis, but not without pain, and a remarkable sense of weakness in the joint. The close coverings may yield so as to allow of slight eversion and slight shortening of the limb. The swelling in these accidents is not likely to be great, unless the surrounding parts are much injured by the blow, or other force, which occasioned the fracture. That which occurs is confined principally to the joint. When these symptoms exist, we might, I think, fairly suspect the existence of a fracture; but, in order to make ourselves more certain, we should examine the limb very attentively. This should be done, however, with the utmost caution." (P. 9.)

As the mode in which the manual examination is conducted might materially influence the result of the case, particular directions are given upon this important point. Crepitus, which must be regarded as the most unequivocal sign of fracture, may or may not be produced.

The author next proceeds to inquire into the manner in which the parts are nourished, both before and after the accident, and to point out the difference between a fracture of the cervix femoris within the synovial capsule, when unaccompanied with laceration of the close coverings, and a fracture of the middle of the bone, as far as it regards the reparative process. When fracture of the cervix femoris occurs, many of the vessels which, in the natural state, support the pelvic portion of the bone, must necessarily be destroyed. Upon this subject the author makes many very pertinent observations. We are not aware, however, that any thing new is added to the information we previously possessed upon this point.

He next enters upon the question of whether such unavoidable division of the vessels is sufficient to prevent osseous union. He thinks not. Neither does he conceive that the presence of synovia between the fractured surfaces, upon which by various authorities much stress has, we believe, been laid, is sufficient to prevent union by bony deposit. In other situations, it is argued, the presence of synovia between the fractured surfaces is easily overcome, and an osseous union of the injured parts is not prevented.

"It appears to me that, as far as regards the physical or vital condition of the parts, there is only one circumstance which in any way tends to make the prognosis more unfavorable in fractures of the neck of the thigh-bone within the synovial capsule, when the close coverings are but little injured, than in fractures extending into other joints, which is the division of the arteries that go to the head of the bone, through the substance of the neck; and this is not, in my opinion, sufficient to render it physically impossible that union should take place." (P. 22.)

From the view, in fact, which Mr. Amesbury takes of the subject, the cause of want of union in such cases is not physical, but mechanical: "the fault lies at the door of surgery, and not in the nature of the accident." He fears that the close coverings of the head of the bone, the periosteum and reflected membrane, through which nourishment must principally be conveyed to the head of the femur, are sometimes lacerated after the accident, either by the incaution of the patient or want of skill in the surgeon. This has, indeed, been observed by several surgeons. If, from whatever cause it may happen, the periosteum and reflected membrane are injured after the infliction of the injury, the case is then converted into the state of a fracture very unfavorable for bony union, in which, from the first, the close coverings of the bone are nearly or quite divided.

To this more perplexing kind of accident Mr. A. now directs his attention. He points out the causes and symptoms of these fractures. Even in such cases he does not despair of success; and he therefore stands opposed to many high authorities, whose doctrines he critically examines at some length.

"If (he says) the treatment be good, I am inclined to believe a sufficient number of facts, to prove that union cannot generally be produced in these cases, when properly managed, will never be obtained. I have been led to this opinion by the consideration that, though the periosteum and reflected membrane be completely divided, and the head of the bone consequently deprived of the principal part of the nourishment naturally sent to it, a vascular communication is again speedily reestablished by the production of organized matter, which, after a time, has the resemblance of ligament connecting the fractured ends together. This ligamentous substance is often found extending, in broad bands, from the head of the bone to that portion of the neck which is attached to the trochanters or to the capsular ligament, with which it becomes firmly united. This soft substance also frequently exists between the fractured surfaces, filling up, in a great measure, the breach of bony continuity; and thus, at the same time that it prevents their further separation, it assists in conveying nourishment to the pelvic portion. Where this soft union has taken place, it is probable that the head of the bone is nearly, perhaps quite as well, nourished as when a fracture exists without laceration of the periosteum and reflected membrane, before any other vascular connexion is established between the fractured surfaces than that which is given by these coverings. And, therefore, if this be granted, as far as regards the supply of blood to the pelvic portion, fractures with laceration very soon become placed under circumstances nearly as favorable for the production of callus as where the close coverings are not much torn; a fact which, it appears, has been altogether overlooked by the supporters of non-union; and one which strongly militates against the opinion that want of nourishment in the head of the bone is the principal cause of these fractures having hitherto been so seldom found united." (P. 60.)

The author, in short, infers, that the physical condition of the fractured ends, upon which Sir Astley Cooper founds his principal argument in favor of the opinions he entertains, is not in itself sufficient to account for the hitherto almost constant occurrence of non-union when the fracture is within the capsule of the hip-joint. He does not consider absorption of the cervix femoris as a necessary consequence of fracture of the part. He would rather attribute the shortening of the neck of the bone, which so frequently takes place, to irritation occasioned in the injured parts by frequent motion and unnatural pressure, which none of the usual modes of practice is calculated to prevent.

"If, then, I am right in the opinion that absorption of the cervix femoris is not a necessary result of a fracture of this part, but is commonly produced in consequence of the irritation which is kept up in the joint, especially by that portion of the neck which is attached to the shaft, it will be seen that, if the fractured surfaces were brought together and kept at rest, such irritation would not take place; and, therefore, that absorption of the neck might be prevented by any contrivance capable of fixing the broken ends of the bone, so as to prevent them from moving upon each other. According to this view of the subject, we might conclude that absorption of the neck of the bone is probably referrible to the imperfection of the treatment usually resorted to as a remote cause, and not to any law of nature peculiar to this part." (P. 69.)

As it is probable, Mr. A. contends, that bony union might frequently be produced, fractures of the neck of the femur should not be abandoned till proper means have been employed.

"From what I have myself observed, and from what I have been able to collect from the observations of others, I am led to believe that the causes of non-union, in fractures of the description above mentioned are four, three of which have been noticed by Sir A. Cooper: 1st, A diminution in the quantity of blood sent to the pelvic portion of the bone; 2d, absence of continued pressure; 3d, want of apposition; and 4th, want of rest. The first of these is inseparable from the nature of these cases, but, in my opinion, is to be regarded as the least important; for, though this will have an influence in retarding the uniting process, as I have above stated, I believe that there is now sufficient proof to lead to the conclusion that it is not in itself sufficient to prevent osseous union from being accomplished. The other three causes are referrible to the treatment; and if it can be shewn that we are in possession of mechanical means, by the judicious employment of which, apposition, pressure, and rest might be maintained for any length of time, these three causes, it is evident, will be at once removed; and, as union by bone now and then takes place notwithstanding the operation of these three causes, we might, it seems to me, reasonably expect that it will be very generally produced where the treatment is such as to prevent their occurrence." (P. 75.)

It is confessed that even from the best treatment success cannot be insured. But before the author could become an advocate for the practice of abandoning fractures of the cervix within the capsule, he would very properly require that those surgeons who support it should prove that no advantage is to be derived from mechanical means. They must also shew that there are unequivocal symptoms by which a fracture of this part of the bone can be distinguished, in every instance, from one external to the joint. This he conceives not to be the case.

Pursuing the argument, Mr. A. further remarks, that if fractures within the capsule could not be united by osseous matter, there is no reason why the opposing ends should not be connected by a layer of short ligament, under proper treatment. The advantages of such an union are pointed out. A great object in such cases is to have the ligament as strong as possible; and for this purpose it will be necessary to confine the patient nearly, or quite, as long as would be requisite to effect union by bone.

In the next section, many good practical observations are offered upon fractures of the neck of the femur external to the capsule, without considerable laceration of the periosteum; and, in section 5, Mr. Amesbury considers the same kind of injury to the bone, *with* great laceration of the surrounding parts.

When the fracture is not attended with laceration, the symptoms are so similar to those which are observed when the bone is broken within the capsule, without much injury to the close coverings, that it will be frequently found difficult to distinguish them. The best surgeons may here fail in their diagnosis. When there *is* great laceration of the surrounding parts, the symptoms are much more strongly marked. The eversion and retraction of the limb are very evident. The degree of shortening is usually proportionate to the degree of laceration. In this variety of fracture, however, it is commonly from half an inch to an inch; seldom exceeding an inch. We have also generally considerable tumefaction of the surrounding parts, with ecchymosis. This very rarely occurs when the fracture is entirely within the synovial membrane.

Fractures external to the capsule are occasionally accompanied with *inversion* of the foot. In these cases the fracture of the cervix has been found complicated with fracture of the trochanter. Mr. Guthrie has detailed an instance which tends to illustrate the causes which give rise to this position of the limb. The following is Mr. Guthrie's case:*

" Sarah Gibson, æt. ninety, fell, on the 9th January, from a high stool on which she was sitting, upon the left hip, and, being a heavy woman, suffered considerable injury. I saw her two days afterwards, with Mr. Dillon, of Judd-street, and found the marks of considerable contusion having been sustained by the part, which was very painful and swelled. The limb was rather more than half an inch shorter than the other, and the great toe turned inwards in a manner sufficiently marked, although not quite so decidedly as in a case of dislocation. The limb was moveable in every direction, but these motions were attended by considerable pain, and it could be easily extended to the same length as the other. No crepitus could be distinguished. The patient died on the 22d February, forty-four days after the accident; and, on dissection, a fracture was discovered external to the capsular ligament. The little trochanter was broken off, and with it the attachment of the psoas and iliacus muscles. The head and neck of the femur were separated from the shaft by a diagonal fracture, extending from the upper and outer part of the trochanter major to the trochanter minor, so as to leave the insertions of the pyriformis, gemellus, obturator externus and internus, and quadratus, with the head and neck of the bone. The gluteus medius formed a bond of union at the upper part of the trochanter major, between the broken pieces retaining them in contact. The capsular ligament was not injured, and no steps whatever appeared to have

* Med.-Chir. Transactions, vol. xiii.

been commenced to repair the mischief which had been committed." (P. 95.)

The causes, symptoms, &c. of fractures of the trochanter major, are described in the next section. The prognosis in such cases is favorable: whether attended with a fracture of the neck of the bone or not, they usually unite; and, where there is not comminution, the author believes, with proper management, union may be effected without deformity of the limb. Now and then the injury sustained is so great as to destroy life, especially in old people.

Chapter ii. *Treatment of Fractures of the upper third of the Thigh-bone.*—Before the author points out the mode of practice peculiar to himself, he passes in review the various means ordinarily employed by other surgeons; each of which he deems, if not objectionable, at least insufficient for the completion of the intent for which they are used. For, although the deformity in some instances is not great, cases of union without deformity are rarely seen.

First, of the treatment of fractures of the cervix femoris.—After the active inflammation is reduced, medicines internally are not required, except with a view to remove disease, or to maintain the health of the patient. Aperients, topical bleedings in moderation, and evaporating lotions, will be required to diminish inflammation. The principal part of the treatment is mechanical, and this is the same whatever may be the situation or nature of the fracture. The time necessary for the completion of osseous union will vary.

"The indications to be answered in the mechanical part of the treatment of fractures of the cervix femoris, whether within or external to the joint, are, 1st, to keep the limb of its natural length; 2d, to keep the limb in the bent position; 3d, to prevent eversion or inversion of the foot; 4th, to keep the trochanter a little raised; 5th, to keep the fractured surfaces in close apposition; 6th, to prevent the fractured surfaces from moving upon each other." (P. 114.)

We must pass over Mr. Amesbury's critical examination of the practice of Desault, Boyer, Sir Astley Cooper, Earle, &c. He has attempted to supply the deficiencies in the ordinary modes of treatment by constructing an apparatus which he employs in the management of fractures of the upper part of the thigh-bone, and for various other purposes.

"This apparatus I call a fracture-bed, which, when properly fitted up, consists of a frame which has a joint near the middle, and which is made to support four pieces of board, long enough, when connected, for an adult to rest upon in the extended position; of a foot board, pelvis strap, mattress, and a convenience to receive the feces. The two pieces of board which form the middle plane are made to slide upon each other, so that this plane might be adapted and fixed by screws attached to it, with the greatest accuracy, to the natural length of the patient's thighs. In this plane there is an opening of a form and size to receive the receptacle for the feces. When the receptacle is in the hole, it is retained in its proper position by a shelf, which shuts up so as to close the opening when the receptacle is removed. This plane is connected to the upper and lower planes by rule joints; which allow the three planes to be placed in connexion upon the frame, at any angles that might be required. The joint formed by the middle and upper planes rests upon the middle of the frame when the bed is used. The upper plane may be kept raised from the frame, so as to incline towards the foot of the bed, by means of a supporter appended to its under surface. The loose ends of this supporter are received in racks formed in the upper end of the sides of the frame. At the lower end of this plane the pelvis strap is attached. The lower end of the lower plane is received in racks formed in the sides of the lower end of the frame, which support it so as to make it form with the middle plane any angle that the surgeon might deem advisable. The foot board, which is connected to the lower end of this plane, answers the double purpose of retaining the foot in its proper position, and of keeping the bed-clothes from pressing unpleasantly upon the toes. The mattress consists of two portions, which are sown together, so as to form a joint like that of a palliasse. The part upon which the trunk rests is double the thickness of that upon which the lower extremities are placed." (P. 132.)

In several important particulars, Mr. A. remarks, this fracture-bed differs from Mr. Earle's. The particular mode in which it is to be applied is minutely described. Cases are also detailed illustrative of the facility with which fractures of the neck of the thigh-bone may be managed by the use of it.

Excepting in a few minor particulars, the treatment of fractures of the trochanters major and minor, which is next discussed, does not differ from the same kind of injury of the neck of the femur.

Part II. *Fractures of long standing.*—Such cases are met with at all periods of life. The causes of non-union are constitutional, constitutional and local jointly, or purely local. Amongst the former may be enumerated various diseases greatly disturbing the system, natural debility, or acquired by debauchery, &c. In pregnancy, there is frequently much disturbance of the whole system, which may either retard or prevent the union of a fracture. The local causes are many, such as disease in the bone, want of apposition, diminished action from the improper continuance of cooling or sedative lotions, want of rest.

Again Mr. Amesbury feels himself called upon to disapprove of the ordinary practice. " I should say that the cause of death in nine cases out of ten, resulting after compound fractures and compound dislocations, might be fairly attributed to the inadequacy of the mechanical treatment." This is, indeed, a very sweeping reprehension, and we hope and trust that, in the author's zeal for improvement, he has a little exaggerated the evils he purposes to remedy. He justly asserts, that if the broken ends of bones are displaced by the involuntary action of the muscles, it is the fault of the treatment: and yet this is a very common excuse for deformity.

When fractures of long standing are examined, the ends of the bone are sometimes found connected together by a structure resembling ligament, or by a ligamento-cartilaginous structure. Sometimes the fractured ends are enlarged from a deposition of bone around their edges, and are connected together by a preternatural capsule, containing a fluid resembling synovia.

Having animadverted upon the different modes of treatment commonly adopted for the purpose of producing an union in fractures of long standing, which it is well known are generally unsuccessful, and frequently unsafe, the author details the plan he has himself employed with advantage. It consists principally in skilfully applying local pressure and rest. For the detailed account of it we must refer to the work itself, where its superiority is made very manifest by its striking success in many cases which are related, and in which other means had either totally or partially failed, even under the hands of justly celebrated surgeons.

The volume concludes with some practical observations upon the treatment of fractures of long standing, which do not admit of being united by the influence of pressure and rest alone.

Every practical surgeon must be much interested in the perusal of Mr. Amesbury's book. If it should be thought that he sometimes too unreservedly canvasses the opinions and treatment of his chirurgical brethren, let it be remembered that, if his "phrase is bold, his arguments are strong."

